# Optical Characterization of Materials for Precision Reference Spheres for Use with Structured Light Sensors

**DOI:** 10.3390/ma16155443

**Published:** 2023-08-03

**Authors:** Pablo Zapico, Victor Meana, Eduardo Cuesta, Sabino Mateos

**Affiliations:** Department of Construction and Manufacturing Engineering, Campus of Gijon, University of Oviedo, 33204 Gijon, Spain; zapicopablo@uniovi.es (P.Z.); ecuesta@uniovi.es (E.C.); sabino@uniovi.es (S.M.)

**Keywords:** structured light, material optical characterization, non-contact digitizing, precision spheres, point cloud filtering

## Abstract

Traditionally, 3D digitizing sensors have been based on contact measurement. Given the disadvantages of this type of measurement, non-contact sensors such as structured light sensors have gained the attention of many sectors in recent years. The fact that their metrological performance is affected by the optical properties of the digitized material, together with the lack of standards, makes it necessary to develop characterization work to validate materials and calibration artifacts for the qualification and calibration of these sensors. This work compares and optically characterizes different materials and surface finishes of reference spheres used in the calibration of two structured light sensors with different fields of application, with the aim to determine the most suitable sphere material–sensor combination in each case. The contact measurement system of a CMM is used as a reference and, for the processing of the information from the sensors, the application of two different filters is analyzed. The results achieved point to sandblasted stainless steel spheres as the best choice for calibrating or qualifying these sensors, as well as for use as registration targets in digitizing. Tungsten carbide spheres and zirconium are unsuitable for this purpose.

## 1. Introduction

The development of scanning sensors capable of recording three-dimensional (3D) characteristics of the objects around us has become a key issue considering that the physical world in which we move is 3D. In the field of engineering, these sensors have changed the paradigm of product verification, replacing inspection processes based on the use of measuring instruments with processes consisting of digitizing for coordinate measurement.

Traditionally, digitizing has been carried out using contact measurement systems mounted on machines of various architecture called Coordinate Measuring Machines (CMM). These sensors, which are connected to a stylus usually ending in a small sphere, both with high rigidity and hardness, are responsible for detecting the instant at which the end of the stylus contacts the scanned surface. Thus, the 3D position of the contacted surface point can be recorded. Given their high performance when evaluated against metrological standards, these types of probes are often used as metrological reference systems for calibration, measurement, or characterization of other measurement sensors. However, they have a series of disadvantages: their point capture rates are low, they can show accessibility problems when digitizing objects with complex geometry, and they are difficult to integrate into the production chain. In addition, although they can be considered non-destructive inspection techniques, they must contact the surface of the object and their use is not advisable in flexible or soft materials, as well as in environments that require high levels of cleanliness.

Nowadays there are a multitude of sensors capable of non-contact 3D scanning, overcoming many of the drawbacks of contact measurement. Relevant advances in recent decades in the development, manufacture, and miniaturization of imaging and digital processing devices have made this possible. The principle of operation of these sensors is varied, light interferometry [1], time-of-flight [2], stereo vision [3], focus variation [4], laser triangulation [5], and structured light [6], among others. Many disciplines other than product inspection, such as medicine, architecture, art, security, topography, or human–machine interaction have found them of interest. In terms of product inspection, and specifically in dimensional and geometric verification, laser triangulation sensors mounted on CMMs have been the most interesting option for decades as they have shown metrological performance close to that of contact measurement systems [7,8,9,10]. However, the main drawback is the need for a CMM, which is complex, almost always not portable, and requires a high degree of expertise and a controlled environment. With structured light sensors, the scenario is different. They can be used to obtain digitized images without the use of any external positioning equipment. The advantages this brings have led to an increasing interest in these sensors in recent years. Applications for in-line or in-process product verification [11,12,13,14], as well as for general inspection tasks on products of various shapes, sizes, and locations [15,16,17,18] are the most typical ones.

The basic structured light system consists of the camera–projector pair. The camera captures different patterns emitted by the projector that are projected onto the surface of the object to be digitized. The cameras commonly used are charge-coupled devices (CCD) or complementary metal-oxide-semiconductors (CMOS). The patterns used usually consist of monochromatic or different-colored visible stripes [19], or in near-infrared light invisible to the human eye [20]. There is also the possibility of using different types and sizes of these fringes depending on the application and the degree of accuracy required [21,22]. By processing the images captured with the camera by different methods, it is possible to detect the distortion of the patterns on the surface of the object and, consequently, to convert this information into 3D point clouds [23,24]. Today, structured light sensors equipped with multiple cameras and projectors are being developed to overcome the limitations shown by camera–projector pairs in terms of occlusion problems and capture speed in the case of large or complex surfaces [25].

As occurs with other non-contact digitizing sensors, such as laser triangulation ones [26,27], the performance of structured light sensors is affected by certain optical issues. These are related both to the characteristics of the digitized surface [28,29,30,31,32,33,34], i.e., material, finish, color, transparency, brightness, geometry, special properties (as shown by plasmonic materials), certain coatings, etc., and to the environment [35], i.e., ambient illumination [36], light refraction, etc. Although in most cases manufacturers provide information on the metrological accuracy of these sensors, the fact is that the artifacts used, including material and finish, the procedure followed, and the accuracy results do not usually follow any recognized standard, given the lack of such standards. In addition, the information provided by the manufacturers regarding the test conditions is non-existent or not very detailed. This has led to the development of recent works seeking to determine the metrological performance of these sensors [37,38,39,40] by comparing their results with that of other metrological systems placed upstream in the metrological chain. Despite the aforementioned influence of material and artifact finish on the results of structured light sensors [28,29,30,31,32,33,34,35,36], most of these works use a single type of material, ignoring the influence it may have on the sensor results. Some choose materials with scanner-friendly surfaces without further discussion [37], while others quite rightly analyze the effects of spheres’ material on the accuracy of scan data [39] in order to decide on the most appropriate artifact material for their sensors. However, they do not discuss the influence of the finish or analyze the application of specific filtering for each sphere material. Additionally, it is important to highlight the lack of studies comparing sensors with different fields of application and, above all, with different kinds of operation: stationary sensors versus handheld sensors.

The aforementioned facts highlight the need to carry out characterization work to identify materials, finishes, and filtering methods that allow a better evaluation with more guarantees of the metrological performance of structured light sensors with different fields of application and with different types of operation. This will make it possible to calibrate them under traceable conditions and to reliably define their field of application. To this end, it is advisable to use metrological artifacts with a proven performance, made of different materials with different optical characteristics that can affect digitizing and using procedures analogous to those applied to other metrological systems. This includes artifacts made of primitive geometries, such as planes or spheres, similar to those used in the verification of CMMs [41], as well as materials with high hardness, high microstructural stability, and low thermal expansion [42] such as WC or ZrO_2_, among others.

In this work, different materials with different surface finishes are characterized in order to determine the most suitable for use as calibration artifacts of different types of structured light sensors. The sensors studied have different fields of application and different scanning procedures. There is also a big difference in their cost. On the one hand, Einscan-SP by Shining 3D is a low-cost sensor for the automatic scanning of small objects with high accuracy requirements. During scanning, this sensor remains static. On the other hand, Leo by Artec 3D, which is more expensive, is a professional sensor designed for manual scanning of objects of various sizes and with lower accuracy requirements.

Commercial precision spheres made of different materials and surface finishes are used as reference artifacts for the evaluation of both sensors. These spheres are intended for the calibration of coordinate metrology equipment [41] and reverse engineering. Additionally, for digitized oriented systems, these spheres can also be used as registration targets in digitizing large objects or groups of objects that require high accuracy [43,44,45,46].

After measuring the spheres with the contact measurement system of a CMM, in order to have reference values, they were digitized with both sensors, i.e., Einscan-SP and Leo. The resulting point clouds were processed with a self-developed algorithm that performs a least squares fit to the sphere geometry. This allowed, on the one hand, evaluation of the quality of the point clouds captured with each sensor by analyzing their residuals and their point density. On the other hand, it made it possible to evaluate the quality of cloud reconstruction by comparing the results of diameter and form error with respect to the reference values obtained by contact with the CMM. In the analysis of this quality, the application of two filters was studied, one based on the standard deviation of the residuals and the other based on the angular coverage of the point clouds. This work shows the influence of the material on the performance of the sensors, the relevant differences between the performance of each of them, as well as the need to perform specific filtering for each sensor and material combination. In this context, optical characterization of the sphere materials was carried out, allowing recommendations to be made on the suitability of the sensor–sphere material as well as on the specific filtering conditions for each case. The conclusions drawn from this work allow users of this type of sensor to know what type of sphere material to use and under what filtering conditions to calibrate or qualify their sensor. Additionally, when they need spheres as registration targets in digitizing large objects of complex geometry.

## 2. Materials and Methods

### 2.1. Materials

In this work, the six spheres listed in Table 1 were used as artifacts made of different materials and with different finishes. These spheres are commercial spheres specially designed for metrological equipment verification. Their diameters are between 20 and 25 mm and can be classified according to the type of material: tungsten carbide (WC), ceramics (Ce-i), coated steel (Co), and sandblasted stainless steel (Sb). As shown in the photographs presented in Table 2, this collection of spheres comprises different colors and different types of finish (Table 1). The coated sphere, Co-1, is a calibration specific to a HP-L-10.6 sensor (Hexagon, Stockholm, Sweden) based on laser triangulation. Its coating material is unknown as it is a trade secret of the supplier.

**Figure 1:**
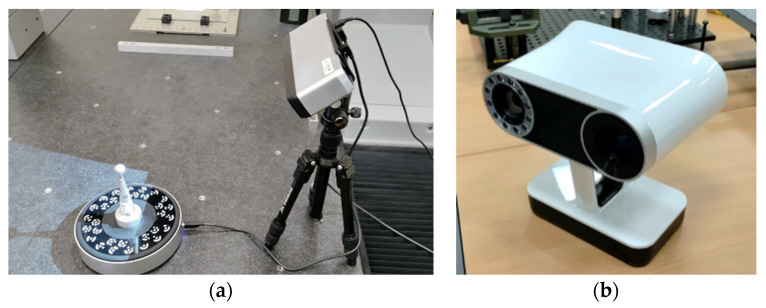
Structured light sensor analyzed: (**a**) Einscan-Sp by Shining 3D, (**b**) Leo by Artec 3D.

Two non-contact scanning sensors based on the structured light technique were analyzed in this study. On the one hand, a low-cost sensor aimed at generalist scanning, Einscan-SP (Shining 3D, Stuttgart, Germany) [48], Figure 1a. This sensor has a head equipped with a white light projector and two cameras capable of capturing textures. In addition, for objects of small size and weight (Table 3), it is equipped with a turntable that allows the object to be oriented with respect to the sensor without the need for operator intervention. To completely digitize the external surface of an object, it is necessary to obtain captures from different points of view at a rate of 4 s per capture, Table 3. In each of these captures, in which the sensor and the object must have static positions, a partial point cloud of the object’s surface is obtained. These partial clouds can then be aligned and merged to compose a single cloud of the object’s entire surface. To physically set up the necessary viewpoints, in the case of digitizing objects compatible with the turntable, the turntable’s automatic rotation can be used. For large or heavy objects, it is necessary to manually move the object or sensor, although it should be noted that the sensor must be stationary at the time each snapshot is captured.

The control of the entire system, which includes a projector, cameras, and turntable, is carried out from the EinscanTool application supplied by the manufacturer. This application runs on a computer connected via USB to the sensor head, which must be plugged into an electrical outlet. The alignment and merging process of the point clouds can also be performed from the same control application. For this, the system allows different options [49] depending on whether the turntable has been used or not and depending on whether it is desired to align with the geometric characteristics of the digitized object or to use digitized targets. In the case of not using the turntable, the point clouds can be aligned by detecting geometric features of the object in several of these clouds (feature alignment), or by using digitizing targets previously attached to the surface of the object before digitizing (markers alignment). On the other hand, if the turntable is used, in addition to the previous two options, the alignment can be performed with a preset angular rotation of the turntable (turntable alignment), or with the targets coded on the turntable surface itself (turntable-coded target alignment). If the digitized object has symmetry or regular shape (prisms, cylinders, spheres, etc.) the manufacturer recommends using the markers alignment method or the turntable coded target alignment method. The latter is more practical and reliable as it uses the turntable-attached targets (Figure 1a), which are external to the object body [49].

The other structured light sensor analyzed is a Leo (Artec 3D, Luxembourg) [50], shown in Figure 1b, whose main features are presented in Table 3. This professional sensor is designed as a compact and fully portable digitizing unit. It is equipped with a structured light system using VCSEL as a light source, a processing unit based on the NVIDIA^®^ JetsonTM platform, a battery, an operator interface display, and several sensors, i.e., accelerometer, gyroscope, and compass. This configuration, together with its ergonomics and low weight, allows large volumes to be scanned by manually moving the sensor around the digitized object. All the processing of the captured information is carried out by the device and a preview of the point cloud can be obtained, practically in real time. The point cloud includes texture information which, together with the information from the different sensors of the device, facilitates the process of aligning the captured information.

**Table 3:** Einscan-SP [48] and Leo [50] information.

Spec	Units	Einscan-SP	Leo
Camera resolution	MP	1.3	2.3
Light source	-	White light	3D-VCSEL ^a^, 2D-white light
Net weight	kg	4.2 ^b^	2.6
Working distance	mm	290–480	350–1200
Point accuracy	µm	50 ^c^	100 + 300·L/1000 ^d^
Max. scan volume	mm	200 × 200 × 200 ^e^	244 × 142 ^g^
	mm	1200 × 1200 × 1200 ^f^	838 × 488 ^h^
Single scan time	s	4	0.023
Manufacturer	-	Shining 3D	Artec 3D
Price	EUR	3000	34,800
Cloud generation	-	By external computer	By the device

^a^ Vertical-Cavity Surface-Emitting Laser; ^b^ including turntable; ^c^ at single shoot; ^d^ L being in mm; ^e^ with turntable (up to 5 kg parts); ^f^ without turntable; ^g^ closest range; ^h^ largest range.

On the other hand, a DEA Global Image Coordinate Measuring Machine (CMM) was used to assess the metrological quality of the reconstruction of the spheres fitted from the point clouds captured with the structured light sensors. This CMM has a Renishaw PH10-MQ indexed head and an SP25M scanning touch probe, coupled with a 30 mm long ceramic stylus ended in a 4 mm diameter ruby sphere. The metrological performance of this machine according to ISO 10360 [41] is given in Equations (1) and (2). The Computer-Aided Inspection (CAI) software PC-DMIS 2018 R2 (Hexagon, Stockholm, Sweden) was used to control this machine.

(1) $$R_{0,MPL}=2.2\;µm$$

(2) $$E_{0,MPE}=2.2+3L\cdot 10^{-3}\;µm,L\;in\;mm$$

Finally, regarding the point cloud processing software, on the one hand, Geomagic Control X 2020 (3DSystems, Rock Hill, SC, USA) software was used to eliminate parts of the tooling or the environment that could have been captured from the point clouds of the spheres. On the other hand, different routines were implemented in Matlab R2022a (The MathWorks, Natick, MA, USA) for the processing and representation of the data captured with the structured light systems and the CMM.

### 2.2. Methods

**Figure 2:**
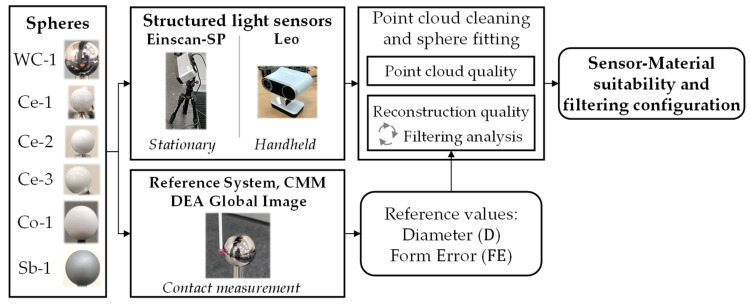
Brief representation of the methodology applied in the work.

The methodology applied in this work is briefly summarized in Figure 2. Firstly, the spheres were calibrated in the CMM using several measurements of 25 contact points homogeneously distributed in the upper hemisphere of each one of them, as recommended by ISO 10360. During the calibration process, the room temperature was controlled at the range of 20 ± 1 °C. The CAI software was used to extract the diameter and the form error of each sphere. The form error is defined as the radial difference between the outermost point and the innermost point considering the best-fit sphere carried out by the CAI software.

After measurement in the CMM, the spheres were digitized using both structured light sensors. In the case of the Einscan-SP, each sphere was digitized individually using the turntable. In these scans, the spheres were mounted in the center of the turntable using a specific tool. During the digitizing, captures were taken in 12 orientations homogeneously distributed in the 360° of rotation of the turntable. From each of these orientations, a point cloud was obtained and later merged with the rest using the turntable coded target alignment method, given the geometrical characteristics of the sphere [49]. In this way, a single point cloud was obtained for each sphere. Before analyzing the point clouds of the different spheres, they were cleaned using Geomagic software. Points captured during digitizing that belonged to external parts, such as support tooling or the turntable, were removed. After this, the cleaned clouds were exported to XYZ files in ASCII format.

For digitizing the spheres with the Leo sensor, the same support tooling was used as with the Einscan-SP. While the spheres remained in static positions, they were digitized by manually moving the sensor. Unlike the scanning with the Einscan-SP, all the spheres were digitized in a single point cloud. In this process, the movement of the sensor and the time spent on each sphere were as similar as possible. After digitizing, the Leo sensor directly provided an entire point cloud that contains all the spheres. Using Geomagic software, the fragments of this cloud corresponding to each sphere were isolated and then cleaned and exported in the same way as the Einscan-SP clouds.

Once the cleaned point clouds of the spheres captured with both sensors were available, they were imported into a specifically developed Matlab routine. This routine allows the fitting of a point cloud to a sphere using the least squares method. For this adjustment, we start from the equation of the sphere (3) and isolate the parameters $x_{0},\;y_{0},\;and\;z_{0}$, which represent the center of the sphere, and the parameter $x_{0}^{2}+y_{0}^{2}+z_{0}^{2}-R^{2}$, which allows us to determine the radius of the sphere, R, once its center is known. The calculation of these parameters, vector $\vec{c}$ in Equation (4), can be carried out using the pseudoinverse matrix of A multiplied by the vector $\vec{b}$. This matrix and vector are obtained using the information of the n-points of the cloud of each sphere: $x_{n},\;y_{n},\;and\;z_{n}$. From this calculation, a fitted center $c_{f}=(x_{f},y_{f},z_{f})$ and radius $R_{f}$ are obtained, being the fitter diameter $D_{f}$.

(3) $${\left(x-x_{0}\right)}^{2}+{\left(y-y_{0}\right)}^{2}+{\left(z-z_{0}\right)}^{2}=R^{2}$$

(4) $$A=\left(\begin{array}{cc} \begin{array}{ccc} 2x_{1} & 2y_{1} & 2z_{1}\\ \vdots & \vdots & \vdots \\ 2x_{n} & 2y_{n} & 2z_{n} \end{array} & \begin{array}{c} -1\\ \vdots \\ -1 \end{array} \end{array}\right);\vec{c}=\left(\begin{array}{c} \begin{array}{c} x_{0}\\ y_{0} \end{array}\\ \begin{array}{c} z_{0}\\ x_{0}^{2}+y_{0}^{2}+z_{0}^{2}-R^{2} \end{array} \end{array}\right);\vec{b}=\left(\begin{array}{c} x_{1}^{2}+y_{1}^{2}+z_{1}^{2}\\ \vdots \\ x_{n}^{2}+y_{n}^{2}+z_{n}^{2} \end{array}\right)\to A\cdot \vec{c}=\vec{b}$$

**Figure 3:**
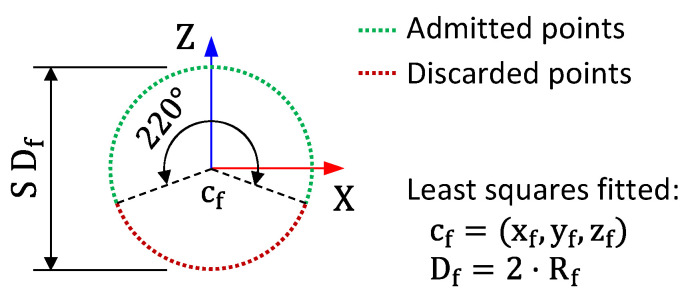
Representation of the angle coverage filter of 220°.

Once the spheres had been adjusted, a filter was applied that discarded cloud points beyond a 220° coverage angle from the pole of the spherical geometry, as illustrated in Figure 3. Given the greater accessibility allowed by the Leo sensor, the original clouds obtained showed a higher coverage angle than the clouds obtained with the Einscan-SP. Therefore, this filter allowed a comparison of the clouds obtained with both sensors under equal conditions.

Once the clouds were trimmed for an angle of 220°, the sphere fit was performed again, and the residuals associated with each of them were determined. These residuals, ${Res}_{i}$, are obtained as the radial distance between each point and the theoretical surface of the sphere obtained in the least squares adjustment (see Equation (5)) where $x_{i},\;y_{i},\;and\;z_{i}$ are the position of each point. On the other hand, the point density for each sphere, PD, was determined as the number of points admitted in the cloud divided by the area covered by the cloud, at this point the equivalent to a covered angle of θ = 220° (see Equation (6)). Both the residuals associated with the different points and the point density of the clouds were used to assess the quality of the clouds.

(5) $${Res}_{i}=R_{i}-R_{f}=\sqrt{{\left(x_{i}-x_{f}\right)}^{2}+{\left(y_{i}-y_{f}\right)}^{2}+{\left(z_{i}-z_{f}\right)}^{2}}-R_{f}$$

(6) $$PD=\frac{nº\;of\;points}{4\pi \cdot R_{f}^{2}\cdot \frac{\theta }{360{}^{\circ}}}\left[Points/mm^{2}\right],\;being\;R_{f}\;in\;mm\;and\;\theta \;in\;degrees$$

Once the quality of the clouds had been analyzed, their reconstruction quality was evaluated. To do so, the deviation of the diameter and the form error of the fitted spheres ($D_{f}$ and $FE_{f}$) with respect to those obtained with the CMM ($D_{CMM}$ and $FE_{CMM}$) were determined. Given the large deviations observed in some cases, the application of two types of filters was proposed to improve the quality of these two parameters. One of these filters was the angle coverage filter, explained above. The other filter is based on applying a coverage factor, k, to the standard deviation observed in the residuals. This filter allows eliminating outlier points that show radius values outside the range $R_{f}\pm k\cdot \sigma$, σ being the standard deviation of the residuals determined previously in the analysis of point clouds quality.

After analyzing the influence of these filters on the reconstruction quality of the point clouds using a configuration of θ = [140°, 180°] for the angle of coverage and k = [2, 3] for the coverage factor applied to standard deviation, each point cloud was processed with the optimal filter configuration. With these data, the possible influence of the material on the quality of the clouds obtained with each sensor was analyzed and recommendations were made.

## 3. Results and Discussion

Table 4 shows the sphere measurement results obtained by means of CMM. As can be seen, the spheres have diameters close to the nominal values except in the case of the coated sphere, Co-1, where the larger real diameter of the sphere can be explained by the coating. As for the form error, it is observed that the spheres have a high geometrical quality, with a form error lower than 5 µm in all cases. The Co-1 sphere presents the highest form error, which is explained by the surface irregularities due to the coating.

**Figure 4:**
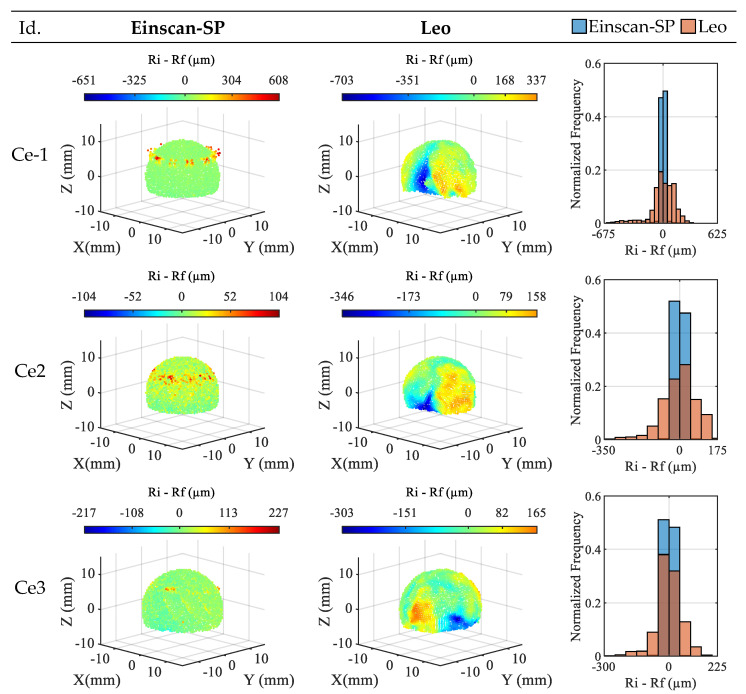
Residuals obtained for the ceramic spheres with the sensors (5× deviation magnification).

At this point it should be noted that for the WC-1 sphere, acceptable digitizing results were not obtained with either of the two sensors studied, so it was excluded from the analysis. Regarding the ceramic spheres, Figure 4 shows the spatial representation and the histogram representation of the residuals obtained in the sphere fitting of the clouds captured with the two sensors. Notice that the spatial representation includes a 5× magnification of the deviation detected for each point of the clouds.

Considering the spatial representation, it is observed that the residual values obtained in the clouds from the Einscan-SP sensor show a more random spatial distribution on the sphere surface than those obtained with the Leo sensor. Although on the Ce-1 sphere, the Einscan-SP cloud shows some concentration around the 40° parallel, related to the direction in which the sensor head was pointing to the sphere at the time of digitizing, the Leo clouds show broad regions of residue concentration where extreme negative and positive values are reached. These regions and the magnitude of these deviations cannot be explained by the form error of the spheres, below 5 µm in all cases (Table 4). This effect is also noticeable in the histogram representations of the residuals (right side of Figure 4), which are more symmetric and centered at zero for the Einscan-SP clouds than for the Leo clouds. As for the ranges of the residuals, no major differences are observed between the two sensors for the Ce-1 and Ce-3 spheres. For the former, 1259 µm versus 1040 µm, and for the latter, 444 µm versus 468 µm, using the Einscan-SP and Leo, respectively. In contrast, notable differences between results with both sensors are observed for the Ce-2 sphere, 208 µm for Einscan-SP versus 504 µm for Leo. Based on the range of the residuals, the Ce-1 sphere is the sphere in which the worst results are obtained for both sensors. As for the best results, for the Einscan-SP they are obtained in the Ce-2 sphere by far, while for the Leo they are obtained for Ce-3 although by little difference with Ce-2.

**Figure 5:**
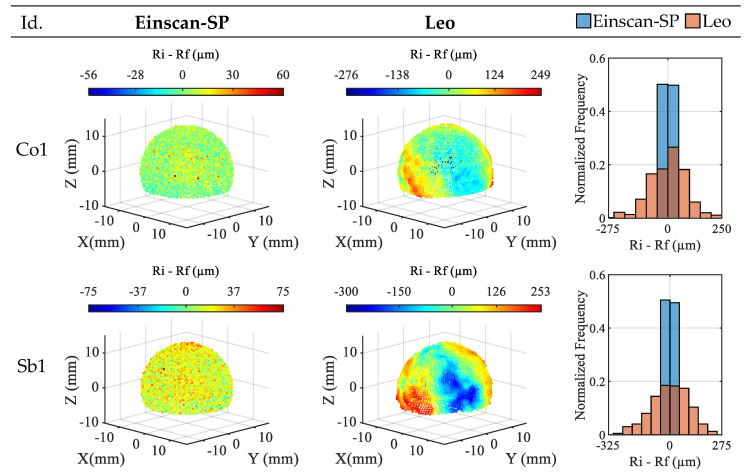
Residuals for the non-ceramic spheres obtained with the sensors (5× dev. magnification).

Similar representations of the residuals for the Co-1 and Sb-1 spheres are shown in Figure 5. As in the case of the ceramic spheres, the Einscan-SP clouds show residuals with more random spatial distribution and more symmetric histograms with respect to zero than those obtained by Leo.

Once again, the clouds obtained with the latter show areas of concentration of residuals of a large magnitude not explained by the form error of the spheres (see Table 4). The residual ranges obtained in these spheres are 116 µm versus 525 µm for the Co-1 sphere and 150 µm versus 553 µm for the Sb-1 sphere, using Einscan-SP and Leo, respectively. In the case of Einscan-SP, these ranges are narrower than those obtained for the ceramic spheres. In the case of Leo, the ranges are around the narrowest ones obtained in the ceramic spheres (Ce-2 and Ce-3). The non-ceramic sphere in which the best residuals, i.e., those with the narrowest range, are obtained with both the Einscan-SP and the Leo is the coated one, Co-1, by more difference in the former sensor, 34 µm, than in the latter, 28 µm. Furthermore, in the case of the Einscan-SP, the Co-1 sphere is where the best results are obtained among all the spheres used, although for this sensor the difference between the Co-1 and Sb-1 residuals is small.

**Figure 6:**
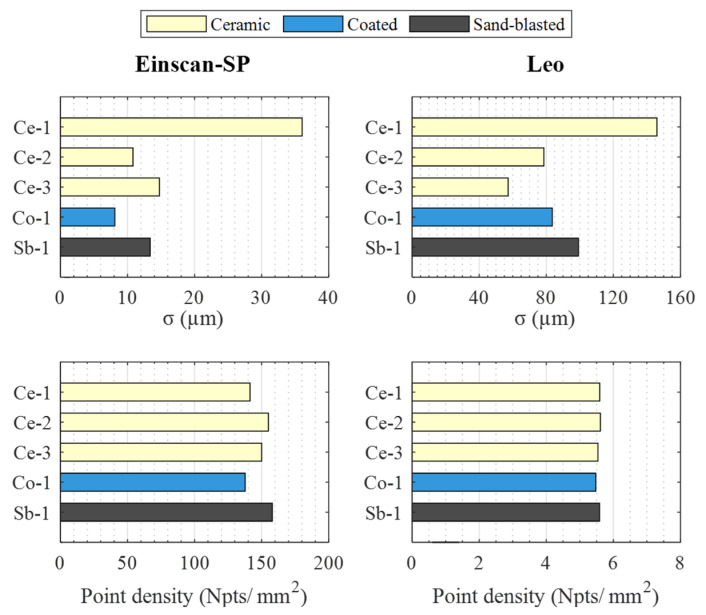
Standard deviations (**top**) and point densities (**bottom**) obtained with the sensors: Einscan-SP (**left**), Leo (**right**).

Figure 6 shows the standard deviation and the point density of the clouds obtained for the different spheres with each sensor. In the case of the standard deviation, the Einscan-SP captures clouds with lower residuals dispersion than the Leo, between 8 µm and 36 µm for the former, and between 55 µm and 145 µm for the latter. This indicator also corroborates that the sphere with the worst results with both sensors is Ce-1, while the sphere with the best for the Einscan-SP is Co-1, and with the Leo is Ce-3.

Regarding point density, it is observed that the clouds captured with the Einscan-SP are much denser than those obtained with the Leo, above 130 points/mm^2^ in all spheres with the former, compared to about 5 points/mm^2^ with the latter. In the Einscan-SP clouds, differences of up to 20 points/mm^2^ are observed between the different spheres, while in the Leo clouds these differences are practically negligible. In the case of the Einscan-SP, for which these differences in density are appreciable, no clear correlation is detected between the density of points and the quality observed in the analysis of the residuals. Both for the sphere in which the best residuals were detected, Co-1, and for the sphere in which the worst residuals were detected, Ce-1, a very similar density of points is obtained, around 140 points/mm^2^.

**Figure 7:**
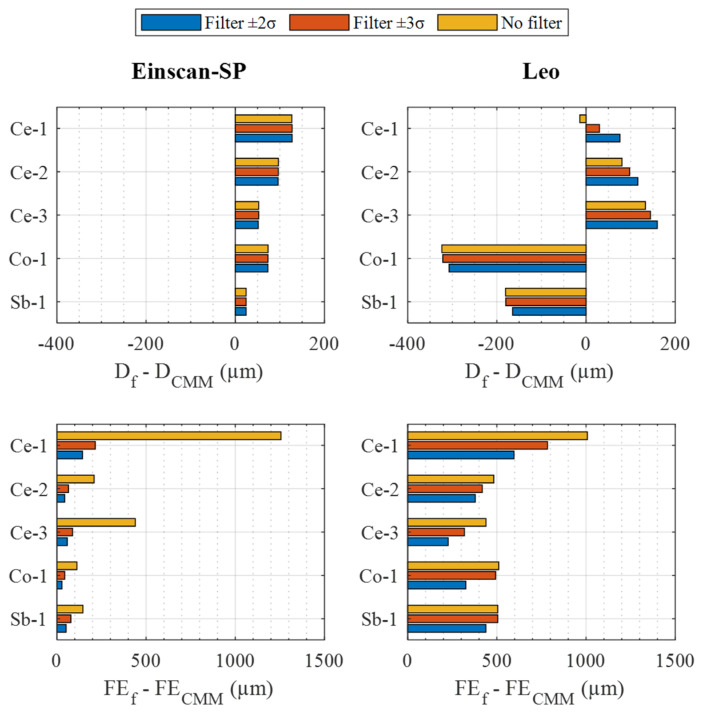
Diameter (**top**) and form error (**bottom**) deviations regarding CMM measurement for the two sensors related to the k·σ filter value: Einscan-SP (**left**), Leo (**right**).

To analyze the reconstruction quality of the spheres achievable with both sensors, two filters were applied to reduce the differences between the results obtained with the sensors and the CMM in terms of diameter and form error (see Table 4). Figure 7 shows the deviations of these parameters as a function of the k value of the applied standard deviation coverage filter. 

Among these results, looking at those obtained under unfiltered conditions, a couple of questions are worth asking. On the one hand, regarding the diameter, Einscan-SP overestimates it in all cases, while Leo overestimates it for two spheres (Ce-2, Ce-3) and underestimates it for the other three (Ce-1, Co-1, Sb-1). The magnitude of these deviations is lower for Einscan-SP than for Leo, except in the case of the Ce-1 and Ce-2 spheres. On the other hand, regarding the form error, it is observed that the values obtained with both sensors are far from those measured in the CMM, between 112 µm and 1256 µm, due to the noise shown by the point clouds. The magnitude of these deviations for the Einscan-SP compared to those of the Leo is higher in Ce-1, quite similar in Ce-3, and lower in the Ce-2, Co-1, and Sb-1 spheres.

Analyzing the influence of the application of the coverage filter, different behavior is observed depending on the parameter, i.e., diameter deviation or form error, and the sensor–sphere combination. On the one hand, in the case of the diameter deviation and the Einscan-SP sensor, practically no alteration in the value of this parameter is observed due to the application of the filter. On the contrary, in the case of the Leo sensor, an influence in all the spheres is noticed, although in different ways depending on the sphere. For this sensor, not applying the filter on the ceramic spheres (Ce-1, Ce-2, Ce-3) and applying it with k = 2 on the non-ceramic spheres (Co-1, Sb-1) allows us to obtain the smallest diameter deviation with respect to the CMM. On the other hand, in the case of the form error deviation, in both sensors and in all the spheres, a great improvement is obtained, although in a more noticeable way in the Einscan-SP when applying a coverage factor k = 2.

**Figure 8:**
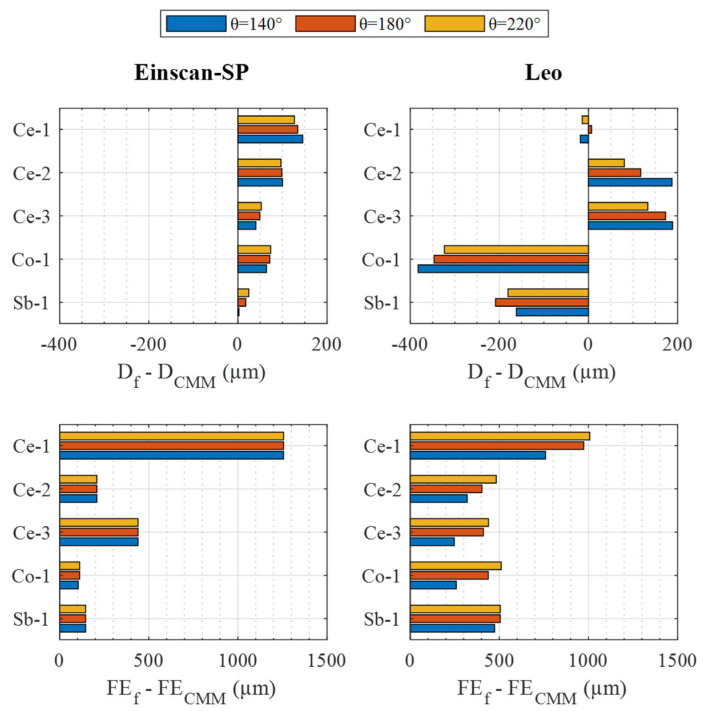
Diameter (**top**) and form deviations (**bottom**) regarding CMM measurement for the two sensors related to the θ filter value: Einscan-SP (**left**), and Leo (**right**).

Figure 8 shows an analogous representation as a function of the value of the angular coverage filter applied. Analyzing the influence of this filter for the case of diameter deviation, in the case of the Einscan-SP, it is observed that it is interesting to use a filtering value of 220° for the Ce-1 and Ce-2 spheres and 140° for the other spheres. In the case of Leo, it is interesting to use 180° for Ce-1, 220° for Ce-2, Ce-3, and Co-1, but 140° for Sb-1. On the other hand, as far as the form error deviation is concerned, in the case of Einscan-SP it is observed that the influence of this filter is marginal. On the contrary, in the Leo this influence is remarkable, with it being the most interesting to use an angle coverage of 140° in all cases.

Considering the different influence of the filters on the different parameters, it is impossible for some sensor–sphere combinations to find a filter configuration that simultaneously optimizes the diameter and form error deviation with respect to those of the CMM. An example of this is the combination Leo sensor and Ce-2 sphere. In this case, as far as the k·σ filter is concerned (Figure 7), the absence of filtering allows us to obtain the smallest diameter deviation, while a k = 3 filtering allows us to obtain the smallest form deviation. Similarly, the absence of filtering in the angular coverage filter (Figure 8) allows us to obtain the smallest deviation in diameter, while a 140° coverage filtering allows us to obtain the smallest deviation in form with respect to the CMM. 

**Figure 9:**
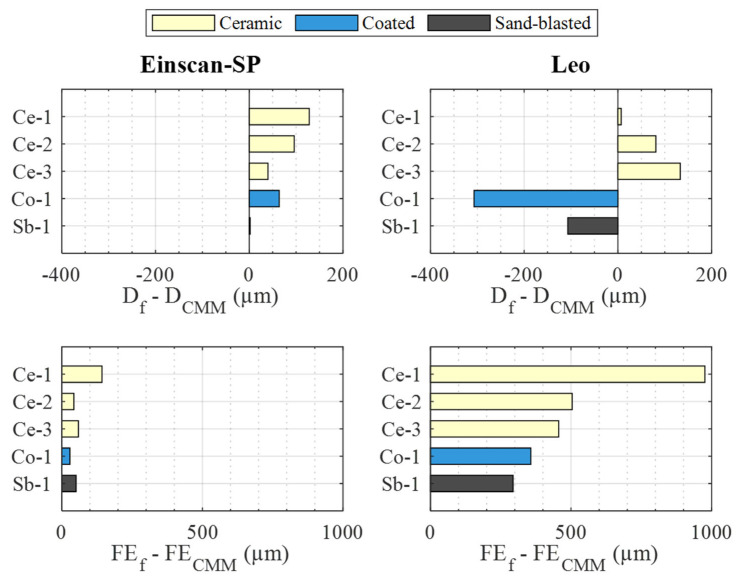
Results with the optimal filtering for each sensor–sphere combination for diameter (**top**) and form error (**bottom**) deviations regarding the CMM results: Einscan-SP (**left**), Leo (**right**).

Taking this casuistry into account, when deciding which filter configuration is the most suitable for each sensor–sphere combination, priority is given to obtaining the smallest possible diameter deviation, followed by the reduction in the form error deviation. This is because a sensor calibration process using a sphere as a reference artifact prioritizes the diameter value detected by the sensor over the form error, although it is also interesting to keep the defect in the detection of the latter under control. Thus, the optimal filtering configuration presented in Table 5 is obtained for each sensor and sphere. As can be seen, in the case of the Einscan-SP, the angle θ is chosen to allow the smallest diameter deviation and a factor k = 2 to allow the smallest form error deviation without practically influencing the diameter. In the case of the Leo, the optimal configuration is reduced to obtaining the smallest diameter deviation, since it is incompatible in many spheres to simultaneously obtain the smallest diameter and form error deviations. In addition to the optimal filter configuration, Table 5 shows the results obtained for these configurations for the different sensor–sphere combinations. These results are also summarized in Figure 9.

On the one hand, as far as diameter deviation is concerned, the Einscan-SP sensor achieves lower deviations in general than the Leo. The former shows deviations between 2 µm and 96 µm, while the latter shows deviations between −307 µm and 133 µm. Only in the case of the Ce-1 and Ce-2 spheres are the deviations obtained with the Leo lower than those obtained with the Einscan-SP, although this difference is reduced in the Ce-2 sphere. Regarding the Ce-1 sphere, the deviation of the Einscan-SP is much higher than that of the Leo. However, the fact that the residuals obtained with both sensors in the case of this sphere had the highest range and showed certain concentrations not explained by the sphere form error, Figure 4, as discussed above, makes it inadvisable to use this sphere with these sensors. Discarding the Ce-1 sphere, the best performance of the Einscan-SP is observed with the Sb-1 sphere and the worst with Ce-2, while for the Leo the best performance is obtained with Ce-2 and the worst with Co-1.

On the other hand, once the Ce-1 sphere is discarded, in terms of form error deviation, the Einscan-SP has a smaller deviation magnitude, between 29 µm and 59 µm, than the Leo, between 294 µm and 504 µm. From the point accuracy of each sensor (Table 3) the expected form error for a theoretically perfect sphere can be determined. To do so, it must be considered that the detectable form error would be twice the point accuracy value since this accuracy can affect whether each point is detected inward or outward from the theoretical sphere surface. Thus, the form errors that would be expected to be detected with the Einscan-SP would be around 100 µm and with the Leo around 206 µm to 208 µm, for the smallest and largest diameter spheres, respectively. As can be seen, the form error deviations with respect to the CMM are within this expected value in the case of the Einscan-SP, being higher in the Leo, although it is closest to the Sb-1 sphere.

## 4. Conclusions

This paper characterizes different materials and surface finishes for their use in the calibration of structured light sensors. Commercial precision spheres were used as calibration artifacts due to their proven reputation as a metrological reference and registration targets in scanning tasks. The structured light sensors analyzed have different fields of application, different types of operation, and differ greatly in price. The Einscan-SP by Shining 3D is a low-cost scanner aimed at the automatic scanning of small objects with high precision, while the Leo by Artec 3D is a more expensive professional scanner aimed at handheld scanning of large objects with lower precision requirements. The performance shown by each sensor for each sphere was analyzed in terms of point cloud quality (spatial distribution of residuals, histograms of residuals, standard deviation, and point density) and reconstruction quality (differences in diameter and form error detected with respect to those measured with a touch probe of a CMM). The application of a residual standard deviation coverage filter and a cloud angular coverage filter were also analyzed. This work demonstrates the different performances of these sensors depending on the material and finish of the spheres, as well as the filtering conditions applied. 

The main finding of the point cloud quality analysis is that the Tungsten Carbine sphere is not suitable for this type of sensor. On the other hand, the Einscan-SP clouds are denser and show more randomly distributed residuals than the Leo clouds, although no correlation was found between higher cloud density and higher residual quality.

Regarding the analysis of the reconstruction quality, it was determined that the Ce-1 sphere, Polished ZrO_2_, is not suitable for use with any of the sensors. On the other hand, it was found necessary to apply the proposed filters particularized for each sphere-sensor material case. Doing it this way, for the Einscan SP the Sb-1 sphere, sandblasted stainless steel, achieves the best results with a slight diameter deviation, 2 µm, and a form error deviation expected for this sensor, 51 µm. It should be noted that Ce-2, Ce-3, and Co-1 spheres could also be used, although with fewer guarantees. As for the Leo, although the smallest diameter deviation is obtained for the Ce-2 sphere, it is advisable to use the Sb-1 sphere also because a slightly higher diameter deviation is observed for this one, but with a form error deviation closer to the expected value, 294 µm obtained versus 208 µm expected. The use of Leo with the rest of the spheres analyzed is discouraged due to the high form errors detected even with the application of the filters.

As future work, it is proposed to apply the methodology of this work to other types of non-contact scanning sensors, i.e., laser triangulation, conoscopic holography, etc., that are influenced by the material or finish of the scanned surfaces. Moreover, once the most appropriate materials and finishes are determined, it is proposed to develop new metrological artifacts to evaluate the volumetric performance of these sensors.

## Figures and Tables

**Table 1:** Information of the spheres used.

Id.	Material	Finishing	Nominal D (mm)
WC-1	Tungsten carbide	Polished	25
Ce-1	ZrO_2_	Polished	20
Ce-2	ZTA ^1^	Matte	20
Ce-3	Al_2_O_3_	Polished	22
Co-1	Coated steel	Coated—Mate	25
Sb-1	Stainless steel	Sandblasted	25

^1^ 10% ZrO_2_ and 90% Al_2_O_3_ by Sandoz [47].

**Table 2:** Photographs and identifiers of the spheres used.

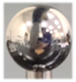	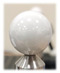	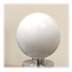	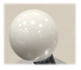	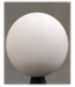	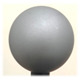
WC-1	Ce-1	Ce-2	Ce-3	Co-1	Sb-1

**Table 4:** CMM sphere measurement results.

Id.	Material	Finishing	CMM Measurement	
			$D_{CMM}$ (mm)	$FE_{CMM}$ (µm)
WC-1	Tungsten carbide	Polished	24.9994	0.4
Ce-1	ZrO_2_	Polished	19.9995	0.8
Ce-2	ZTA	Mate	20.0012	0.8
Ce-3	Al_2_O_3_	Polished	22.0005	1.5
Co-1	Coated steel	Coated—Mate	25.4878	4.6
Sb-1	Stainless steel	Sandblasted	25.0055	2.9

**Table 5:** Optimal filtering for each sensor–sphere combination.

	Einscan-SP				Leo			
	k	θ	$D_{f}-D_{CMM}$	${FE}_{f}-{FE}_{CMM}$	k	θ	$D_{f}-D_{CMM}$	${FE}_{f}-{FE}_{CMM}$
Id.	-	°	µm	µm	-	°	µm	µm
Ce-1	2	220	128	143	0	180	7	976
Ce-2	2	220	96	43	0	220	81	504
Ce-3	2	140	40	59	0	220	133	456
Co-1	2	140	64	29	2	220	−307	357
Sb-1	2	140	2	51	2	140	−107	294

## Data Availability

There is no data shared from this work.
